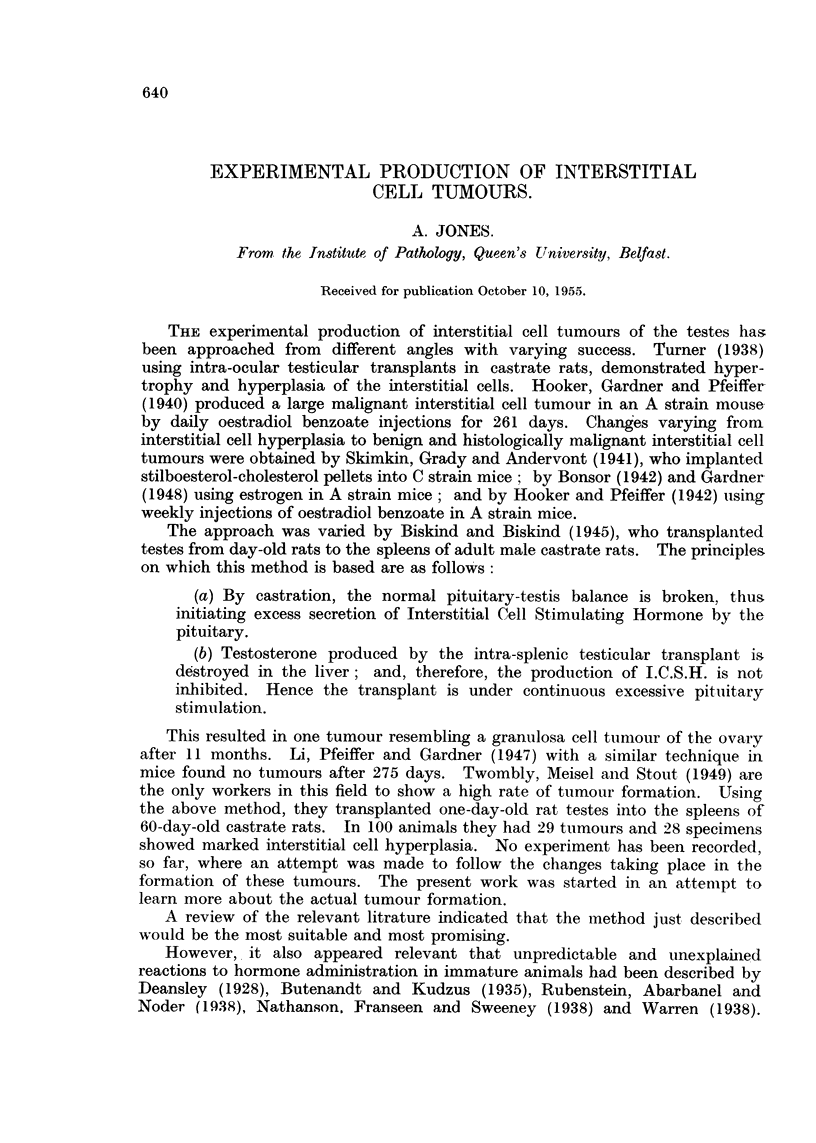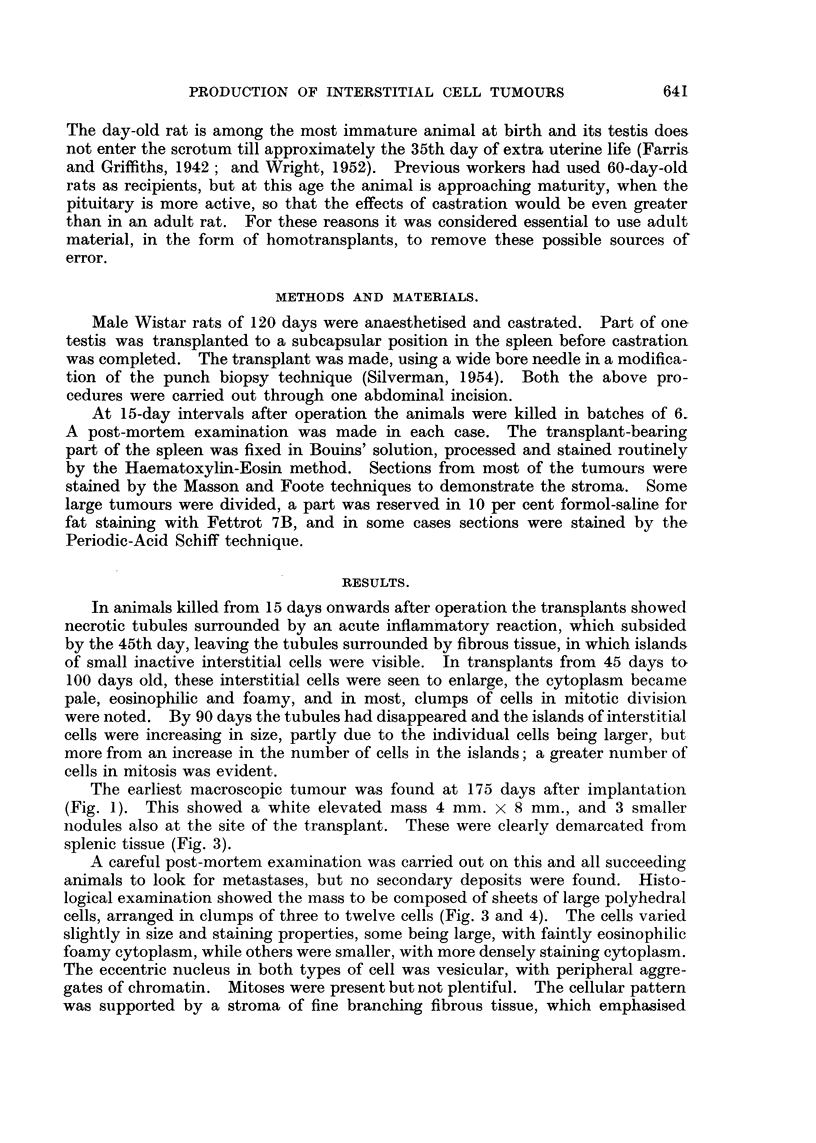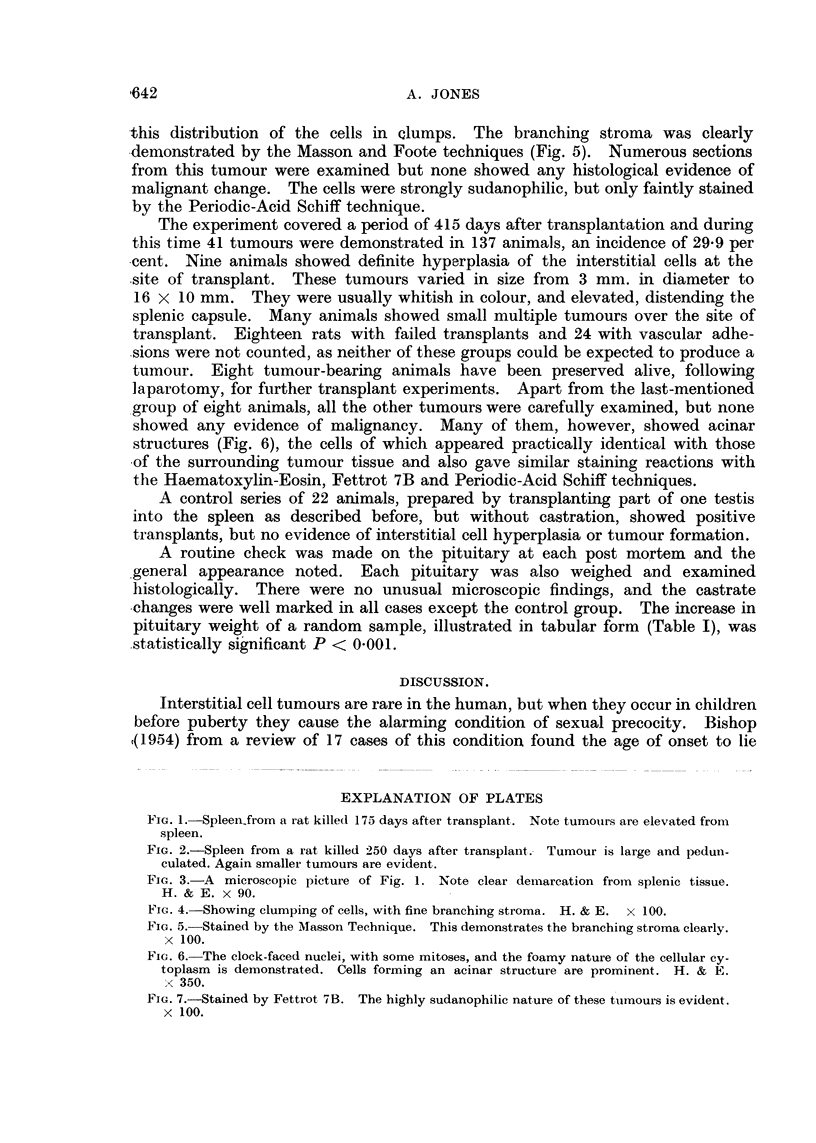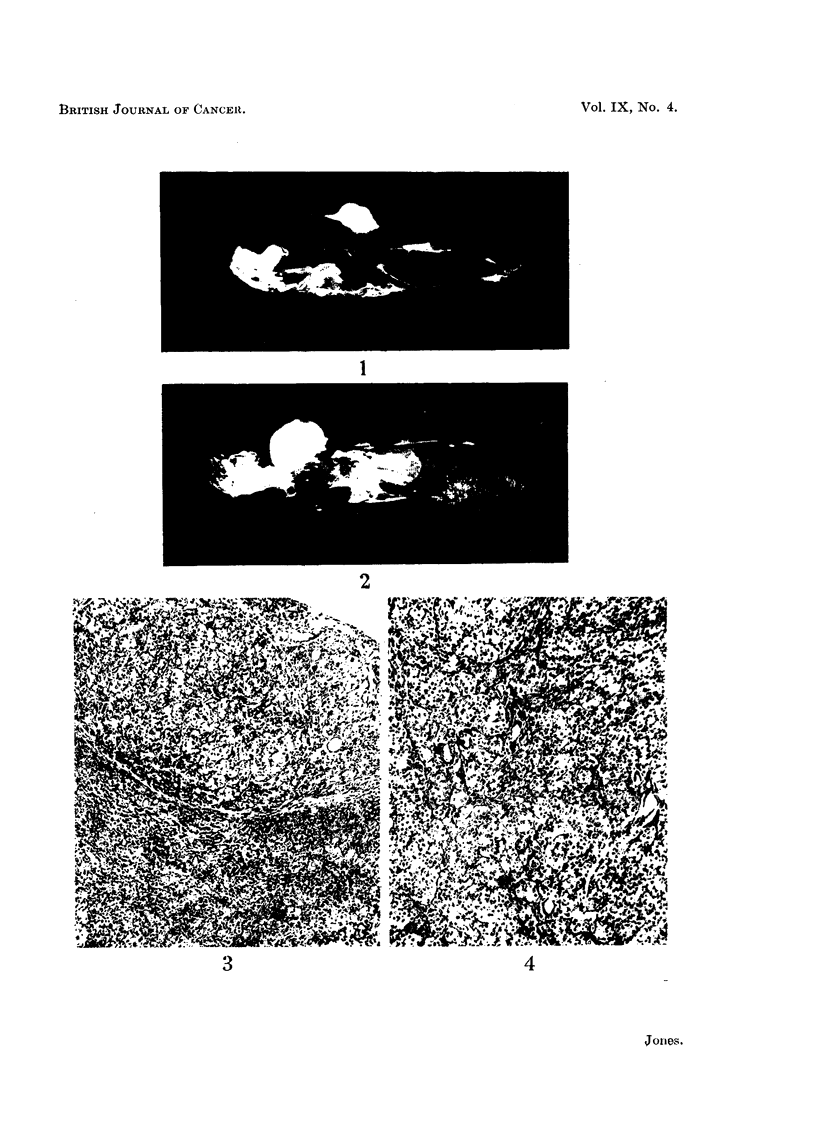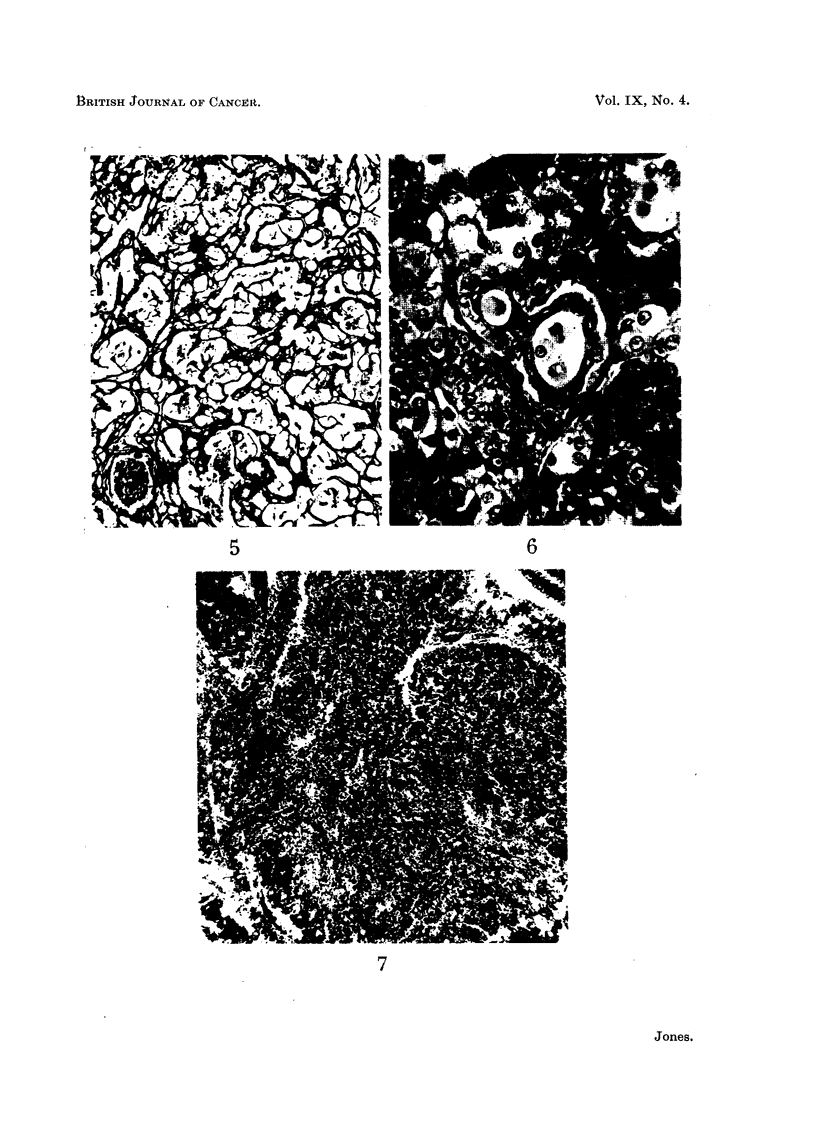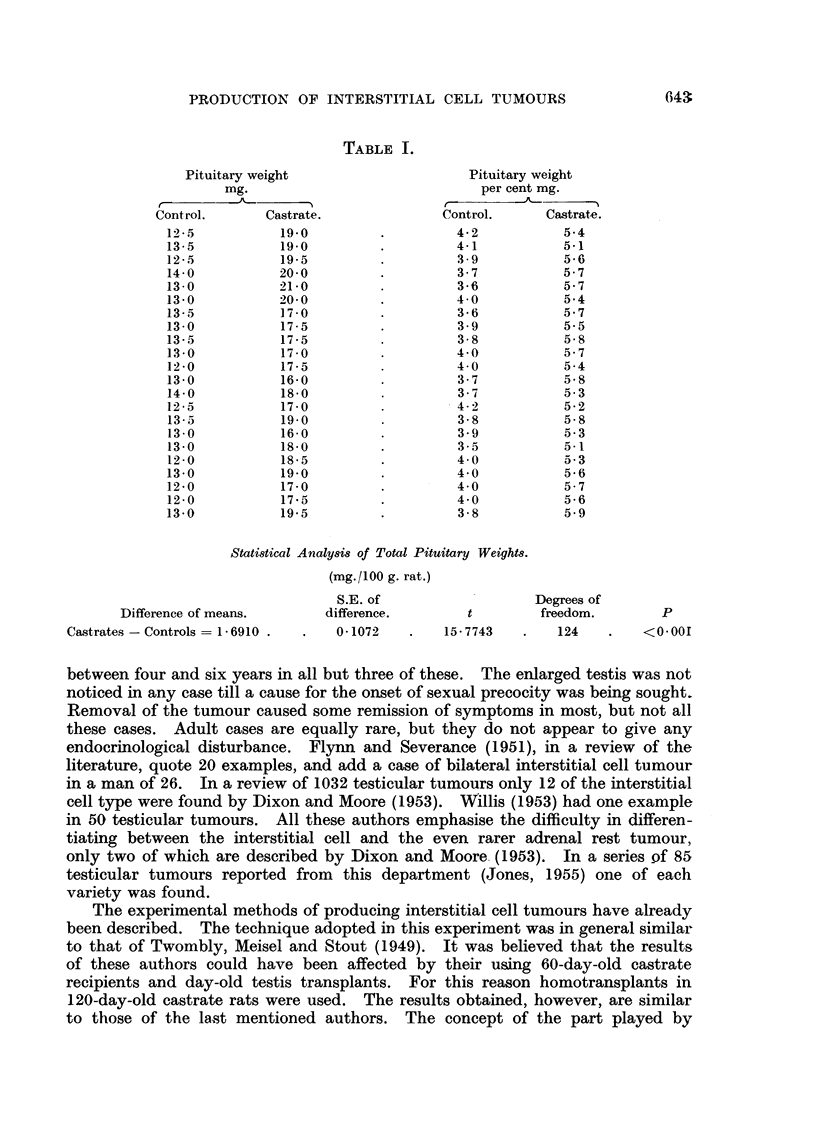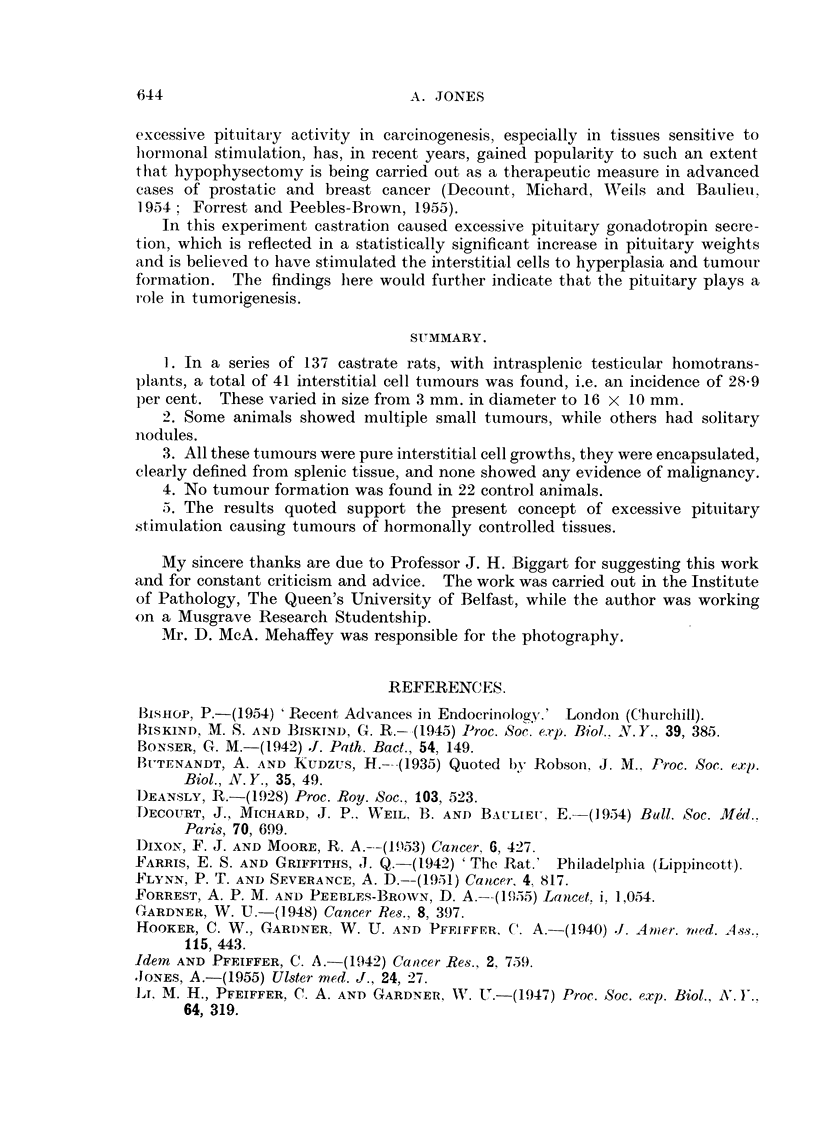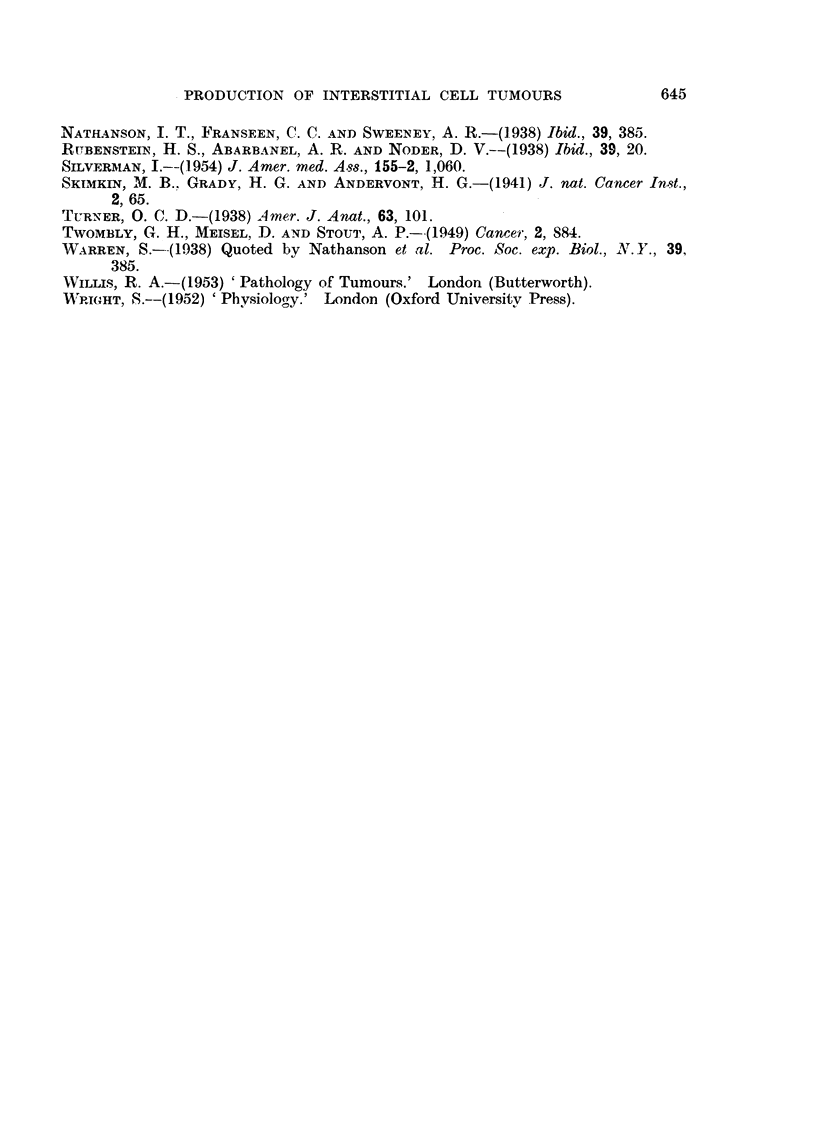# Experimental Production of Interstitial Cell Tumours

**DOI:** 10.1038/bjc.1955.68

**Published:** 1955-12

**Authors:** A. Jones

## Abstract

**Images:**


### EXPERIMENTAL PRODUCTION OF INTERSTITIAL CELL TUMOURS.

A. JONES.

*From the Institute of Pathology, Queen's University, Belfast.*

Received for publication October 10, 1955.

THE experimental production of interstitial cell tumours of the testes has been approached from different angles with varying success. Turner (1938) using intra-ocular testicular transplants in castrate rats, demonstrated hypertrophy and hyperplasia of the interstitial cells. Hooker, Gardner and Pfeiffer (1940) produced a large malignant interstitial cell tumour in an A strain mouse by daily oestradiol benzoate injections for 261 days. Changes varying from interstitial cell hyperplasia to benign and histologically malignant interstitial cell tumours were obtained by Skimkin, Grady and Andervont (1941), who implanted stilboesterol-cholesterol pellets into C strain mice ; by Bonsor (1942) and Gardner (1948) using estrogen in A strain mice ; and by Hooker and Pfeiffer (1942) using weekly injections of oestradiol benzoate in A strain mice.

The approach was varied by Biskind and Biskind (1945), who transplanted testes from day-old rats to the spleens of adult male castrate rats. The principles on which this method is based are as follows :

(*a*) By castration, the normal pituitary-testis balance is broken, thus initiating excess secretion of Interstitial Cell Stimulating Hormone by the pituitary.

(*b*) Testosterone produced by the intra-splenic testicular transplant is destroyed in the liver ; and, therefore, the production of I.C.S.H. is not inhibited. Hence the transplant is under continuous excessive pituitary stimulation.

This resulted in one tumour resembling a granulosa cell tumour of the ovary after 11 months. Li, Pfeiffer and Gardner (1947) with a similar technique in mice found no tumours after 275 days. Twombly, Meisel and Stout (1949) are the only workers in this field to show a high rate of tumour formation. Using the above method, they transplanted one-day-old rat testes into the spleens of 60-day-old castrate rats. In 100 animals they had 29 tumours and 28 specimens showed marked interstitial cell hyperplasia. No experiment has been recorded, so far, where an attempt was made to follow the changes taking place in the formation of these tumours. The present work was started in an attempt to learn more about the actual tumour formation.

A review of the relevant litrature indicated that the method just described would be the most suitable and most promising.

However, it also appeared relevant that unpredictable and unexplained reactions to hormone administration in immature animals had been described by Deansley (1928), Butenandt and Kudzus (1935), Rubenstein, Abarbanel and Noder (1938), Nathanson, Franseen and Sweeney (1938) and Warren (1938).

The day-old rat is among the most immature animal at birth and its testis does not enter the scrotum till approximately the 35th day of extra uterine life (Farris and Griffiths, 1942 ; and Wright, 1952). Previous workers had used 60-day-old rats as recipients, but at this age the animal is approaching maturity, when the pituitary is more active, so that the effects of castration would be even greater than in an adult rat. For these reasons it was considered essential to use adult material, in the form of homotransplants, to remove these possible sources of error.

### METHODS AND MATERIALS.

Male Wistar rats of 120 days were anaesthetised and castrated. Part of one testis was transplanted to a subcapsular position in the spleen before castration was completed. The transplant was made, using a wide bore needle in a modification of the punch biopsy technique (Silverman, 1954). Both the above procedures were carried out through one abdominal incision.

At 15-day intervals after operation the animals were killed in batches of 6. A post-mortem examination was made in each case. The transplant-bearing part of the spleen was fixed in Bouins' solution, processed and stained routinely by the Haematoxylin-Eosin method. Sections from most of the tumours were stained by the Masson and Foote techniques to demonstrate the stroma. Some large tumours were divided, a part was reserved in 10 per cent formol-saline for fat staining with Fettrot 7B, and in some cases sections were stained by the Periodic-Acid Schiff technique.

### RESULTS.

In animals killed from 15 days onwards after operation the transplants showed necrotic tubules surrounded by an acute inflammatory reaction, which subsided by the 45th day, leaving the tubules surrounded by fibrous tissue, in which islands of small inactive interstitial cells were visible. In transplants from 45 days to 100 days old, these interstitial cells were seen to enlarge, the cytoplasm became pale, eosinophilic and foamy, and in most, clumps of cells in mitotic division were noted. By 90 days the tubules had disappeared and the islands of interstitial cells were increasing in size, partly due to the individual cells being larger, but more from an increase in the number of cells in the islands; a greater number of cells in mitosis was evident.

The earliest macroscopic tumour was found at 175 days after implantation (Fig. 1). This showed a white elevated mass 4 mm. $\times$ 8 mm., and 3 smaller nodules also at the site of the transplant. These were clearly demarcated from splenic tissue (Fig. 3).

A careful post-mortem examination was carried out on this and all succeeding animals to look for metastases, but no secondary deposits were found. Histological examination showed the mass to be composed of sheets of large polyhedral cells, arranged in clumps of three to twelve cells (Fig. 3 and 4). The cells varied slightly in size and staining properties, some being large, with faintly eosinophilic foamy cytoplasm, while others were smaller, with more densely staining cytoplasm. The eccentric nucleus in both types of cell was vesicular, with peripheral aggregates of chromatin. Mitoses were present but not plentiful. The cellular pattern was supported by a stroma of fine branching fibrous tissue, which emphasised this distribution of the cells in clumps. The branching stroma was clearly demonstrated by the Masson and Foote techniques (Fig. 5). Numerous sections from this tumour were examined but none showed any histological evidence of malignant change. The cells were strongly sudanophilic, but only faintly stained by the Periodic-Acid Schiff technique.

The experiment covered a period of 415 days after transplantation and during this time 41 tumours were demonstrated in 137 animals, an incidence of 29·9 per cent. Nine animals showed definite hyperplasia of the interstitial cells at the site of transplant. These tumours varied in size from 3 mm. in diameter to 16 × 10 mm. They were usually whitish in colour, and elevated, distending the splenic capsule. Many animals showed small multiple tumours over the site of transplant. Eighteen rats with failed transplants and 24 with vascular adhesions were not counted, as neither of these groups could be expected to produce a tumour. Eight tumour-bearing animals have been preserved alive, following laparotomy, for further transplant experiments. Apart from the last-mentioned group of eight animals, all the other tumours were carefully examined, but none showed any evidence of malignancy. Many of them, however, showed acinar structures (Fig. 6), the cells of which appeared practically identical with those of the surrounding tumour tissue and also gave similar staining reactions with the Haematoxylin-Eosin, Fettrot 7B and Periodic-Acid Schiff techniques.

A control series of 22 animals, prepared by transplanting part of one testis into the spleen as described before, but without castration, showed positive transplants, but no evidence of interstitial cell hyperplasia or tumour formation.

A routine check was made on the pituitary at each post mortem and the general appearance noted. Each pituitary was also weighed and examined histologically. There were no unusual microscopic findings, and the castrate changes were well marked in all cases except the control group. The increase in pituitary weight of a random sample, illustrated in tabular form (Table I), was statistically significant $P < 0{\cdot}001$.

### DISCUSSION.

Interstitial cell tumours are rare in the human, but when they occur in children before puberty they cause the alarming condition of sexual precocity. Bishop (1954) from a review of 17 cases of this condition found the age of onset to lie

---

### EXPLANATION OF PLATES

FIG. 1.—Spleen from a rat killed 175 days after transplant. Note tumours are elevated from spleen.

FIG. 2.—Spleen from a rat killed 250 days after transplant. Tumour is large and pedunculated. Again smaller tumours are evident.

FIG. 3.—A microscopic picture of Fig. 1. Note clear demarcation from splenic tissue. H. & E. × 90.

FIG. 4.—Showing clumping of cells, with fine branching stroma. H. & E. × 100.

FIG. 5.—Stained by the Masson Technique. This demonstrates the branching stroma clearly. × 100.

FIG. 6.—The clock-faced nuclei, with some mitoses, and the foamy nature of the cellular cytoplasm is demonstrated. Cells forming an acinar structure are prominent. H. & E. × 350.

FIG. 7.—Stained by Fettrot 7B. The highly sudanophilic nature of these tumours is evident. × 100.

1

2

3

4

5

6

7

TABLE I.

| Pituitary weight mg. | | | Pituitary weight per cent mg. | |
|---|---|---|---|---|
| Control. | Castrate. | | Control. | Castrate. |
| 12·5 | 19·0 | . | 4·2 | 5·4 |
| 13·5 | 19·0 | . | 4·1 | 5·1 |
| 12·5 | 19·5 | . | 3·9 | 5·6 |
| 14·0 | 20·0 | . | 3·7 | 5·7 |
| 13·0 | 21·0 | . | 3·6 | 5·7 |
| 13·0 | 20·0 | . | 4·0 | 5·4 |
| 13·5 | 17·0 | . | 3·6 | 5·7 |
| 13·0 | 17·5 | . | 3·9 | 5·5 |
| 13·5 | 17·5 | . | 3·8 | 5·8 |
| 13·0 | 17·0 | . | 4·0 | 5·7 |
| 12·0 | 17·5 | . | 4·0 | 5·4 |
| 13·0 | 16·0 | . | 3·7 | 5·8 |
| 14·0 | 18·0 | . | 3·7 | 5·3 |
| 12·5 | 17·0 | . | 4·2 | 5·2 |
| 13·5 | 19·0 | . | 3·8 | 5·8 |
| 13·0 | 16·0 | . | 3·9 | 5·3 |
| 13·0 | 18·0 | . | 3·5 | 5·1 |
| 12·0 | 18·5 | . | 4·0 | 5·3 |
| 13·0 | 19·0 | . | 4·0 | 5·6 |
| 12·0 | 17·0 | . | 4·0 | 5·7 |
| 12·0 | 17·5 | . | 4·0 | 5·6 |
| 13·0 | 19·5 | . | 3·8 | 5·9 |

*Statistical Analysis of Total Pituitary Weights.*

(mg./100 g. rat.)

| Difference of means. | S.E. of difference. | $t$ | Degrees of freedom. | $P$ |
|---|---|---|---|---|
| Castrates — Controls = 1·6910 | 0·1072 | 15·7743 | 124 | $<0·001$ |

between four and six years in all but three of these. The enlarged testis was not noticed in any case till a cause for the onset of sexual precocity was being sought. Removal of the tumour caused some remission of symptoms in most, but not all these cases. Adult cases are equally rare, but they do not appear to give any endocrinological disturbance. Flynn and Severance (1951), in a review of the literature, quote 20 examples, and add a case of bilateral interstitial cell tumour in a man of 26. In a review of 1032 testicular tumours only 12 of the interstitial cell type were found by Dixon and Moore (1953). Willis (1953) had one example in 50 testicular tumours. All these authors emphasise the difficulty in differentiating between the interstitial cell and the even rarer adrenal rest tumour, only two of which are described by Dixon and Moore (1953). In a series of 85 testicular tumours reported from this department (Jones, 1955) one of each variety was found.

The experimental methods of producing interstitial cell tumours have already been described. The technique adopted in this experiment was in general similar to that of Twombly, Meisel and Stout (1949). It was believed that the results of these authors could have been affected by their using 60-day-old castrate recipients and day-old testis transplants. For this reason homotransplants in 120-day-old castrate rats were used. The results obtained, however, are similar to those of the last mentioned authors. The concept of the part played by excessive pituitary activity in carcinogenesis, especially in tissues sensitive to hormonal stimulation, has, in recent years, gained popularity to such an extent that hypophysectomy is being carried out as a therapeutic measure in advanced cases of prostatic and breast cancer (Decount, Michard, Weils and Baulieu, 1954 ; Forrest and Peebles-Brown, 1955).

In this experiment castration caused excessive pituitary gonadotropin secretion, which is reflected in a statistically significant increase in pituitary weights and is believed to have stimulated the interstitial cells to hyperplasia and tumour formation. The findings here would further indicate that the pituitary plays a role in tumorigenesis.

### SUMMARY.

1. In a series of 137 castrate rats, with intrasplenic testicular homotransplants, a total of 41 interstitial cell tumours was found, i.e. an incidence of 28·9 per cent. These varied in size from 3 mm. in diameter to 16 × 10 mm.

2. Some animals showed multiple small tumours, while others had solitary nodules.

3. All these tumours were pure interstitial cell growths, they were encapsulated, clearly defined from splenic tissue, and none showed any evidence of malignancy.

4. No tumour formation was found in 22 control animals.

5. The results quoted support the present concept of excessive pituitary stimulation causing tumours of hormonally controlled tissues.

My sincere thanks are due to Professor J. H. Biggart for suggesting this work and for constant criticism and advice. The work was carried out in the Institute of Pathology, The Queen's University of Belfast, while the author was working on a Musgrave Research Studentship.

Mr. D. McA. Mehaffey was responsible for the photography.

### REFERENCES.

Bishop, P.—(1954) 'Recent Advances in Endocrinology.' London (Churchill).

Biskind, M. S. and Biskind, G. R.—(1945) *Proc. Soc. exp. Biol., N.Y.*, **39**, 385.

Bonser, G. M.—(1942) *J. Path. Bact.*, **54**, 149.

Butenandt, A. and Kudzus, H.—(1935) Quoted by Robson, J. M., *Proc. Soc. exp. Biol., N.Y.*, **35**, 49.

Deansly, R.—(1928) *Proc. Roy. Soc.*, **103**, 523.

Decourt, J., Michard, J. P., Weil, B. and Baulieu, E.—(1954) *Bull. Soc. Méd., Paris*, **70**, 699.

Dixon, F. J. and Moore, R. A.—(1953) *Cancer*, **6**, 427.

Farris, E. S. and Griffiths, J. Q.—(1942) 'The Rat.' Philadelphia (Lippincott).

Flynn, P. T. and Severance, A. D.—(1951) *Cancer*, **4**, 817.

Forrest, A. P. M. and Peebles-Brown, D. A.—(1955) *Lancet*, i, 1,054.

Gardner, W. U.—(1948) *Cancer Res.*, **8**, 397.

Hooker, C. W., Gardner, W. U. and Pfeiffer, C. A.—(1940) *J. Amer. med. Ass.*, **115**, 443.

*Idem* and Pfeiffer, C. A.—(1942) *Cancer Res.*, **2**, 759.

Jones, A.—(1955) *Ulster med. J.*, **24**, 27.

Li, M. H., Pfeiffer, C. A. and Gardner, W. U.—(1947) *Proc. Soc. exp. Biol., N.Y.*, **64**, 319.

NATHANSON, I. T., FRANSEEN, C. C. AND SWEENEY, A. R.—(1938) *Ibid.*, **39**, 385.
RUBENSTEIN, H. S., ABARBANEL, A. R. AND NODER, D. V.—(1938) *Ibid.*, **39**, 20.
SILVERMAN, I.—(1954) *J. Amer. med. Ass.*, **155-2**, 1,060.
SKIMKIN, M. B., GRADY, H. G. AND ANDERVONT, H. G.—(1941) *J. nat. Cancer Inst.*, **2**, 65.
TURNER, O. C. D.—(1938) *Amer. J. Anat.*, **63**, 101.
TWOMBLY, G. H., MEISEL, D. AND STOUT, A. P.—(1949) *Cancer*, **2**, 884.
WARREN, S.—(1938) Quoted by Nathanson *et al.* *Proc. Soc. exp. Biol., N.Y.*, **39**, 385.
WILLIS, R. A.—(1953) 'Pathology of Tumours.' London (Butterworth).
WRIGHT, S.—(1952) 'Physiology.' London (Oxford University Press).